# Idiopathic Isolated Right Ventricular Cardiomyopathy: A Rare Case Report

**DOI:** 10.1055/s-0043-1764376

**Published:** 2023-03-24

**Authors:** Vivek Mohanty, Shubham Kumar Sharma, Sourabh Goswami, Rahul Gudhage, Surender Deora

**Affiliations:** 1 Department of Cardiology, All India Institute of Medical Sciences, Jodhpur, Rajasthan, India

**Keywords:** idiopathic, right ventricle, cardiomyopathy

## Abstract

Idiopathic isolated right ventricular cardiomyopathy is an extremely rare cause of right ventricular failure. Here, we report a case of 65-year-old male presented with chief complaints of dyspnea, fatigue, and bilateral pedal edema for the last 6 months. On clinical evaluation, grade II/III pansystolic murmur was present in left parasternal area. Investigations and imaging revealed dilated right atrium and ventricle with normal pulmonary artery pressure without any etiology. Magnetic resonance imaging ruled out other common causes of right ventricular cardiomyopathy; thus, the patient was diagnosed as a case of idiopathic isolated right ventricular cardiomyopathy that is a diagnosis of exclusion.

## Introduction

Right ventricle (RV) is usually affected with left ventricle (LV) by inherited or acquired cardiomyopathies. Isolated RV cardiomyopathy is a very rare disease and only few cases have been reported in literature. 1 2 Most of the cases that have been reported in literature are secondary to causes like Ebstein's anomaly, arrhythmogenic right ventricular cardiomyopathy (ARVC), and familial RV cardiomyopathy. 3 Isolated cases have been reported in Takotsubo cardiomyopathy, systemic sclerosis, autoimmune hypothyroidism, and isolated RV noncompaction cardiomyopathy. 4 5 6 7 Here, we highlight a very rare case of idiopathic isolated RV cardiomyopathy and approach to its diagnosis.

## Case Report



**Video 1** Echocardiography and color Doppler revealing dilated RA and RV with severe TR and SEC in RA. RA, right atrium; RV, right ventricle; SEC, spontaneous echo contrast; TR, tricuspid regurgitation;.


**Video 2** Four-chamber view (bright blood sequence) showing grossly dilated RA and RV with severe TR. RA, right atrium; RV, right ventricle; TR, tricuspid regurgitation.


**Fig. 1:**
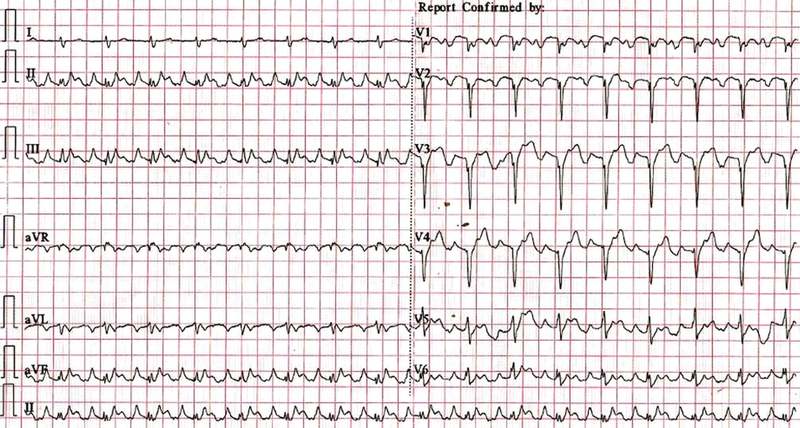
Twelve-lead electrocardiogram showing normal sinus rhythm, right axis deviation, P pulmonale, and poor R wave progression in anterior precordial leads.

**Fig. 2:**
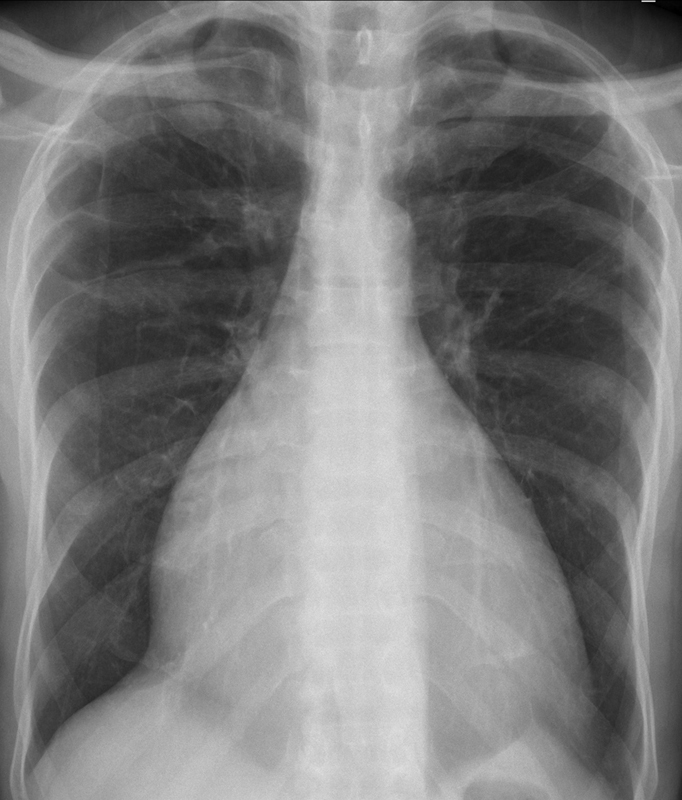
Chest X-ray showing cardiomegaly and normal pulmonary parenchyma.

**Fig. 3:**
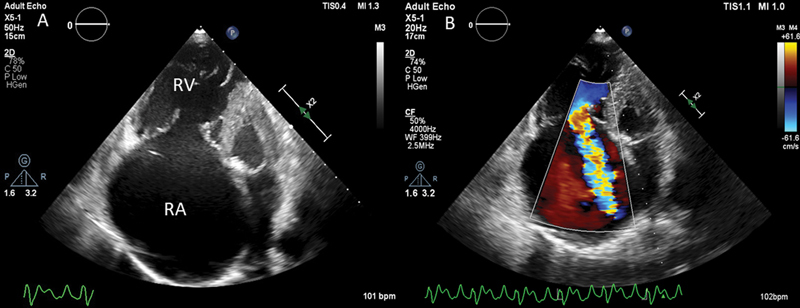
Echocardiogram in modified apical four-chamber view revealing dilated RA and RV ( **A** ). Color Doppler image revealing severe TR ( **B** ). RA, right atrium; RV, right ventricle; TR, tricuspid regurgitation.

**Fig. 4:**
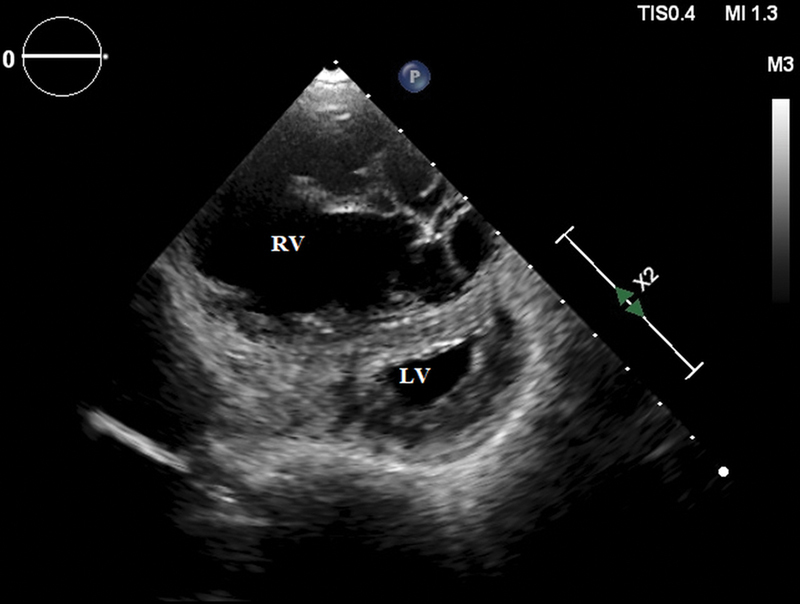
Echocardiogram in parasternal short axis view showing D-shaped LV and dilated RV. LV, left ventricle; RV, right ventricle.

**Fig. 5:**
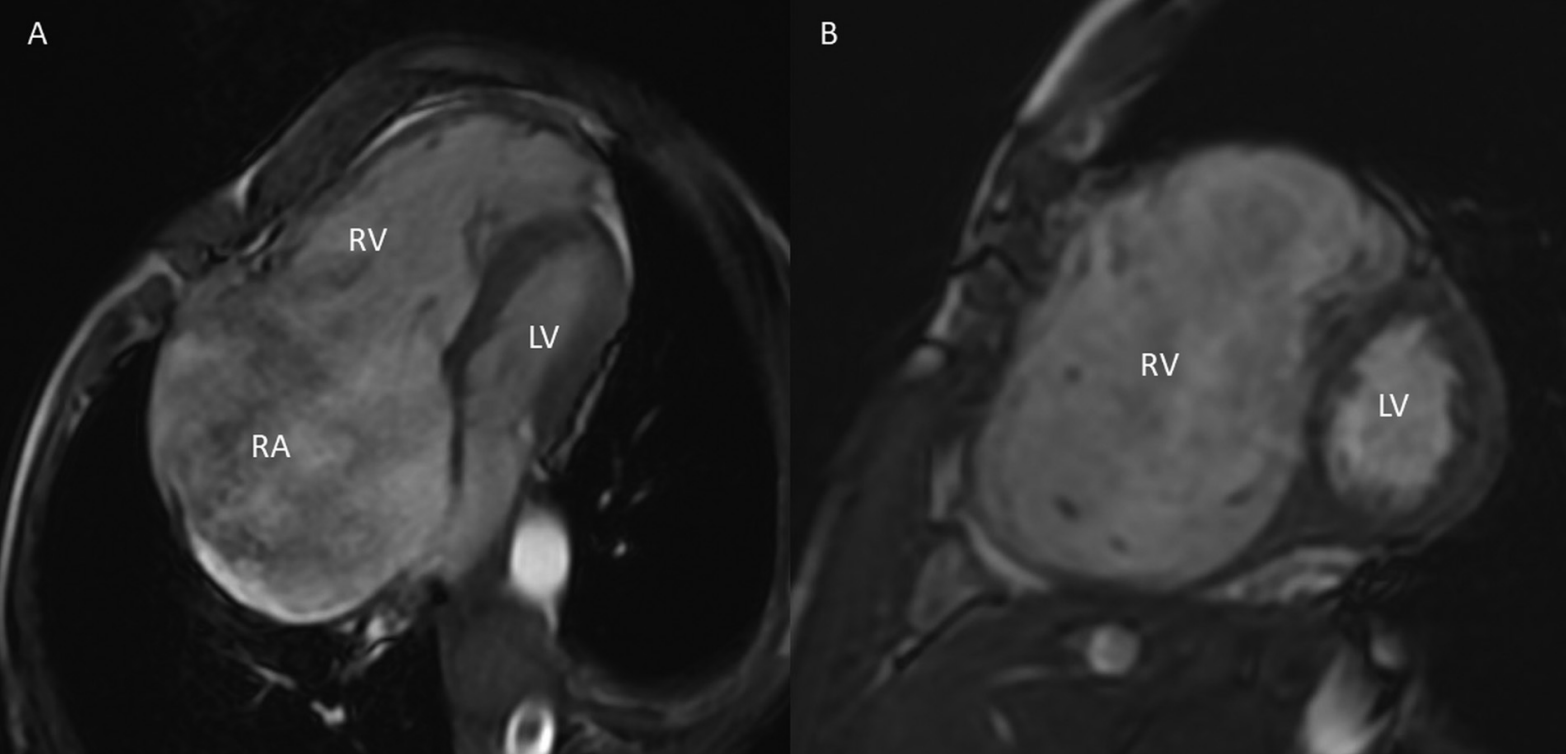
Magnetic resonance imaging showing bright blood sequence in four-chamber view of grossly dilated RA and RV ( **A** ) and in short axis view grossly dilated RV in comparison to LV ( **B** ). LV, left ventricle; RA, right atrium; RV, right ventricle.

A 65-year-old male presented with chief complaints of dyspnea and fatigue on exertion for last 6 months associated with bilateral pedal edema. Cardiovascular examination showed apex beat shifted lateral to the mid-clavicular line and grade II/III pansystolic murmur in left parasternal area that was nonradiating. Electrocardiogram (ECG) showed normal sinus rhythm, right axis deviation, P pulmonale, and poor R wave progression in anterior precordial leads ( Fig. 1 ). Chest X-ray was suggestive of cardiomegaly and normal pulmonary parenchymal and vascular findings ( Fig. 2 ). Transthoracic echocardiography in apical four-chamber view showed dilated right atrium (RA) and RV with severe functional low pressure tricuspid regurgitation (TR) and spontaneous echo contrast in RA ( Fig. 3A , 3B ; Video 1 ). LV cavity was small (D shaped) with normal ejection fraction (left ventricle ejection fraction—55%) ( Fig. 4 ). Pulmonary functions tests and arterial blood gases were also normal. Magnetic resonance imaging (MRI) showed grossly dilated RA and RV (58 mm diastolic diameter) with global RV hypokinesia (right ventricle ejection fraction—21%) and normally attached tricuspid valve leaflets. It also confirmed the functional cause of the severe TR because of ring dilatation of tricuspid annulus (48 mm) ( Fig. 5A and B ; Video 2 ). There was neither fibro-fatty involvement of RV myocardium nor any late gadolinium enhancement. Abnormal venous drainage was also excluded with the MRI. The patient underwent cardiac catheterization that showed normal pulmonary pressures (systolic pulmonary artery pressure/ diastolic pulmonary artery pressure / mean pulmonary artery pressure of 23/8/16 mm Hg, respectively) and mildly raised pulmonary capillary wedge pressure (14 mm Hg). Coronary angiography revealed normal epicardial arteries. The patient was diagnosed as idiopathic isolated form of RV cardiomyopathy and was managed with guideline-directed medical therapy. One-month telephonic follow-up was suggestive of compensated heart failure in New York Heart Association functional class II.

## Discussion

Isolated idiopathic RV cardiomyopathy is very rare disease and is a diagnosis of exclusion. The patients usually present with symptoms of right heart failure as in our case and rarely with syncope. The development of various diagnostic modalities and imaging has helped immensely in evaluation of patients with right heart failure. The ECG in our case revealed poor R wave progression in anterior precordial leads that during initial stage of evaluation has suggested evolved anterior wall myocardial infarction with left ventricular dysfunction as a cause of heart failure. But further diagnostic evaluation by echocardiography suggested dilated RA and RV with clockwise rotation of heart as explanation for poor R wave progression. Previously, most of the patients with diagnosis of isolated idiopathic RV cardiomyopathy were reclassified as ARVC after MRI. Our patient did not meet the Task Force Criteria for ARVC as there was RV dilatation without any area of RV akinesia or dyskinesia or fibro-fatty inflammation. 8 There was also no evidence of epsilon waves in ECG and other features suggestive of the diagnosis of ARVC. Pulmonary cause of RV failure was excluded as pulmonary artery pressure was normal on echocardiography and during cardiac catheterization. The possibility of dysplastic tricuspid valve with secondary RV dilatation was also considered clinically, but the age of presentation and imaging has ruled it out. Isolated noncompaction of RV, a very rare genetic disease, may present with right heart failure, arrythmias, and recurrent pulmonary embolism. 9 The RV has bilaminar structure with compacted and noncompacted segments, dense trabecular meshwork with deep intertrabecular recesses. MRI helps in differentiating it with other differential diagnosis of isolated RV failure. Another differential diagnosis of isolated RV cardiomyopathy is Uhl's anomaly where there is complete absence of RV parietal myocardium and apposition of epicardial and endocardial layers with no adipose tissue in between or signs of necrosis. 10 Most commonly, the patients present in infancy with right-sided heart failure, whereas our patient was elderly male and both echo and MRI revealed normally thickened myocardium. The cardiac biopsy might have helped us to have definitive diagnosis, but the risk of the procedure outweighed its benefit, hence was deferred. Management of isolated RV cardiomyopathy has not been well described in the literature. Symptomatic medical treatment and cardiac transplant are the available management strategies.

## Conclusion

Isolated RV cardiomyopathy is a rare cause of right heart failure and is a diagnosis of exclusion. ECG, chest X-ray, echocardiogram, MRI, and cardiac catheterization may help in excluding the secondary or other genetic causes of RV cardiomyopathy. Guideline-directed medical management and cardiac transplantation are the management strategies for idiopathic RV cardiomyopathy.
